# LC-HRMS and GC-MS Profiling of Urine Free Cortisol, Cortisone, 6Β-, and 18-Hydroxycortisol for the Evaluation of Glucocorticoid and Mineralocorticoid Disorders

**DOI:** 10.3390/biom14050558

**Published:** 2024-05-06

**Authors:** Gregori Casals, María Antonieta Ballesteros, Angielys Zamora, Irene Martínez, Guillermo Fernández-Varo, Mireia Mora, Felicia A. Hanzu, Manuel Morales-Ruiz

**Affiliations:** 1 Biochemistry and Molecular Genetics Department, Hospital Clínic of Barcelona, Institut d’Investigacions Biomèdiques August Pi i Sunyer (IDIBAPS), Centro de Investigación Biomédica en Red de Enfermedades Hepáticas y Digestivas (CIBERehd), 08036 Barcelona, Spain; imartin1@clinic.cat (I.M.); gfvaro@recerca.clinic.cat (G.F.-V.); morales@clinic.cat (M.M.-R.); 2 Department of Fundamental and Clinical Nursing, Faculty of Nursing, University of Barcelona, 08036 Barcelona, Spain; 3 Service of Clinical Analysis, Hospital Universitario Son Espases, 07210 Palma de Mallorca, Spain; antonietabv@hotmail.com; 4 Department of Clinical Biochemistry, Hospital General Universitario Gregorio Marañón, 28007 Madrid, Spain; angielys.zamora@salud.madrid.org; 5 Department of Endocrinology and Nutrition, Hospital Clinic, Institut d’Investigacions Biomèdiques August Pi i Sunyer (IDIBAPS), 08036 Barcelona, Spain; mporta@clinic.cat; 6 Department of Medicine, Faculty of Medicine and Health Science, University of Barcelona, 08036 Barcelona, Spain; 7 Department of Biomedicine, Faculty of Medicine and Health Science, University of Barcelona, 08036 Barcelona, Spain

**Keywords:** urine free cortisol, micro-liquid chromatography, high-resolution mass spectrometry, Cushing’s syndrome, cortisone

## Abstract

Introduction: Urine free cortisol measurements are routinely performed to evaluate hypercortisolism. Despite their analytical inaccuracy, immunoassay-based methods are frequently used. Advances in liquid chromatography–high-resolution mass spectrometry (LC-HRMS) facilitate the incorporation of powerful diagnostic tools into clinical laboratories. In addition to its high analytical specificity and simultaneous analysis of different metabolites, accurate mass measurement allows for untargeted compound identification, which may help to identify clinically relevant metabolites or drugs. Methods: The present study aimed to validate a simple routine LC–HRMS method to quantify cortisol, cortisone, 6β-hydroxycortisol, and 18-hydroxycortisol simultaneously in human urine. Additionally, the study also validated a GC-MS method for the same steroids, evaluated their cross-reactivity with commercial cortisol immunoassays, and quantified the 24 h urine excretion in patients under clinical suspicion or follow-up for hypercortisolism. Results: The LC-HRMS method involved liquid–liquid extraction using dichloromethane, micro-LC for chromatographic separation and detection using the accurate masses of the steroids, and simultaneous high-resolution full scan acquisition. The method presented acceptable linearity, precision, and accuracy. Significant interference from 6β-hydroxycortisol and cortisone was demonstrated in the cortisol immunoassays, which impacted their reliability in the follow-up of patients with hypercortisolism and significant changes in these cortisol metabolites (i.e., due to drug-induced changes in CYP3A4 activity). Conclusion: A rapid and accurate routine LC-HRMS method was validated, which is useful for the evaluation of hypercortisolism and other disorders of glucocorticoid and mineralocorticoid metabolism.

## 1. Introduction

Urine free (unconjugated) cortisol measurements are fundamental in the screening and follow-up of Cushing’s syndrome (CS). Despite their high analytical inaccuracy [1,2], these measurements are widely performed using immunoassay [3,4]. Recent advances in liquid chromatography–mass spectrometry (LC-MS) allow for a considerable reduction in the complexity of the analytical methods, which facilitates the incorporation of powerful diagnostic tools into routine clinical laboratories. In addition to the high analytical specificity that makes them the state-of-the-art techniques for the quantification of steroid hormones, LC-MS allows for the simultaneous analysis of different metabolites, thus providing additional information that may be clinically helpful.

Liquid chromatography–high-resolution mass spectrometry (LC-HRMS) has been associated with qualitative analysis and research, whereas liquid chromatography with tandem mass spectrometry (LC-MS/MS) has been associated with quantitative and routine analysis. However, the currently available LC-HRMS instruments have shown sensitivity and quantitative performance comparable to those of LC-MS/MS for small molecules, including steroids [5,6]. In addition, HRMS provides more accurate masses than triple-quadrupole instruments with a lower resolution. Moreover, current LC-HRMS allows for simultaneous sensitive quantitative and qualitative analyses [5,6,7,8,9]. Thus, high-resolution full scan acquisitions allow for untargeted compound identification, which may be helpful to identify clinically relevant metabolites or drugs. Also, metabolic phenotyping, data mining for biomarker discovery, and retrospective data analysis are advantages for clinical research. These factors, together with good versatility and increasing affordability, explain the increasing interest of clinical and research laboratories in developing analysis methods using LC-HRMS.

The aim of the present study was to validate a simple routine LC–HRMS method to quantify simultaneously the urine concentrations of cortisol, cortisone, 6β-hydroxycortisol, and 18-hydroxycortisol. In addition to the key role of urine cortisol measurements in CS, the cortisol/cortisone ratio (11β-hydroxysteroid dehydrogenase 2 (11βHSD2) activity) is a sensitive marker for the apparent mineralocorticoid excess syndrome [10] and may help to differentiate between Cushing’s disease and ectopic ACTH production [11]. The 6β-hydroxycortisol/cortisol ratio is a widely used endogenous marker for CYP3A activity [12,13,14,15], and 18-hydroxycortisol levels are useful in the evaluation of primary hyperaldosteronism, especially in the diagnosis of its familial forms [10,16].

There are previously validated LC-MS/MS methods for the measurement of urine cortisol and cortisone [17,18,19,20,21,22,23,24,25,26], cortisol and 6β-hydroxycortisol [12,13,14,15,19], and 18-hydroxycortisol [27,28]. Recently, two LC-HRMS urine profiling methods included cortisol, cortisone, and 6β-hydroxycortisol [29,30]. However, to the best of our knowledge, the GC-MS method developed by Shackleton et al. [31] remains the only validated method including cortisol, cortisone, 6β-hydroxycortisol, and 18-hydroxycortisol. Therefore, our study also validated in parallel a GC-MS method based on the one developed by Shackleton et al., which was compared with the LC-HRMS method. In addition, we evaluated the cross-reactivity of the measured cortisol metabolites with commercial cortisol immunoassays and quantified the 24 h urine excretion of the four steroids using LC-HRMS in patients with clinical suspicion of hypercortisolism or in follow-up for CS.

## 2. Materials and Methods

### 2.1. Chemicals Reagents

Cortisol, cortisone, 6β-hydroxycortisol, cortisol-d4, and cortisone-d8 were purchased from Merck (Darmstadt, Germany); the 18-hydroxycortisol was from Steraloids (Newport, RI, USA); the 6β-hydroxycortisol-d4 was from Toronto Research Chemicals (North York, ON, Canada), the 18-hydroxycortisol-d4 was obtained from Cambridge Isotopes Laboratories Inc. (Andover, MA, USA). Methoxyamine hydrochloride, *N*-trimethylsilylimidazole, cyclohexane, dichloromethane, pyridine, and the certified reference material ERM-DA192 were from Merck (Darmstadt, Germany). Mobile phases A and B were obtained from Chromsystems (reference numbers 72011 and 72002, respectively). Ultrapure water was obtained using a Millipore Milli-Q purification system.

### 2.2. Preparation of Stock Solutions, Working Solutions, Calibrators, and Quality Control Samples

Calibration curves were prepared in methanol as a free surrogate matrix. The analytical response differences between urine and methanol were evaluated according to a recovery assessment (Section 2.5.1). Stock solutions of each steroid were prepared at 1 g/L in methanol. Working solution was prepared by mixing and diluting the four metabolites in methanol to a final concentration of each metabolite of 1.25 mg/L. Six-point calibration curves were prepared for the calibration of both the GC-MS and the LC-HRMS methods (12.5, 50, 100, 250, 500, and 1000 µg/L). For the quality controls (QCs), two levels were prepared for both the GC-MS method (25 and 125 µg/L) and the LC-HRMS method (80 and 160 µg/L). The internal standard (IS) stock solutions of cortisol-d4, cortisone-d8, 6β-hydroxycortisol-d4, and 18-hydroxycortisol-d4 were prepared at a concentration of 0.1 g/L in methanol and stored at −20 °C. A combined IS working solution was prepared at a final concentration of 0.010 g/L for each steroid.

### 2.3. GC-MS

#### 2.3.1. Sample Preparation

Prior to the GC-MS analysis, the samples were stored at −20 °C for up to 2 months. The urine samples were centrifuged (3000× *g*, 10 min), and 2 mL was extracted with 5 mL of dichloromethane already containing the combined IS (0.1 µg). After liquid–liquid extraction, the organic phase was evaporated to dryness under nitrogen, and the methyloxime–trimethylsilyl ether derivatives were formed according to two-step derivatization. First, methyloxime formation was achieved by adding 100 µL of a solution of methoxyamine hydrochloride (2% in pyridine) to them and heating them at 55 °C in a thermoblock for 60 min. Second, trimethylsilyl ethers were formed by adding 50 μL of *N*-trimethylsilylimidazole to the oximated substances and irradiating them for 2 min at 600 W in a domestic microwave (SpeedyGrill, Taurus, Oliana, Spain). After derivatization, two microliters were injected into the GC-MS instrument. The same procedure was followed with the calibrators and the QC samples.

#### 2.3.2. Instrumentation

GC-MS analyses were performed using a Shimadzu GCMS-QP2010 Ultra instrument. The final extracts were injected in splitless mode (valve opened at 2 min) into the gas chromatograph interfaced with a mass selective detector. A total of 2 μL was injected into the chromatographic system, and three pre- and post-injection washes (in cyclohexane) were performed between injections. Chromatographic separation was achieved using a Sapiens-5MS+ capillary column (30 m × 0.25 mm internal diameter × 0.25 μm film thickness) from Teknokroma (Barcelona, Spain) with helium as the carrier gas at a constant velocity of 50 cm/s. The temperature program was set to begin at 50 °C, maintained at this temperature for 3 min, elevated at 80 °C min^−1^ to 240 °C, then increased at 2 °C min^−1^ until 290 °C, and finally maintained for 5 min at 290 °C. The total run time was 35 min. The ion source and transfer line temperatures were set to 240 °C and 280 °C, respectively. Following a 22 min solvent delay, the mass detector was operated in synchronous selected ion monitoring (SIM) mode (*m*/*z* 531, 539, 605, 608, 513, 517, 385, 389) using a dwell time of 150 ms. Identification of the analytes in the sample extracts was achieved according to the GC retention time and comparison with the reference standards. Data acquisition and processing were performed using the GCMSsolution workstation software version 4 (Shimadzu, Kyoto, Japan). Figure S1A shows a chromatogram of a urine sample. The retention times were cortisone 25.0 min, cortisol 26.1 min, 6β-hydroxycortisol 26.4 min, and 18-hydroxycortisol 30.7 min.

### 2.4. LC-HRMS

#### 2.4.1. Sample Preparation

Prior to the LC-HRMS analysis, the samples were stored at −20 °C for up to 2 months. After centrifugation, the urine samples (0.5 mL) were extracted with 5 mL of dichloromethane already containing the combined IS (0.03 µg). After liquid–liquid extraction, the organic phase was evaporated to dryness under nitrogen, and the residue was reconstituted in 100 μL of the mobile phases (83% of mobile phase A, 17% mobile phase B) and transferred into an autosampler glass vial. One microliter was injected into the LC-HRMS instrument. The same procedure was followed with the calibrators and the QC samples.

#### 2.4.2. Instrumentation 

Micro-flow LC-HRMS analyses were carried out using a Dionex UltiMate 3000 RSLCnano system coupled to a hybrid quadrupole-Orbitrap mass spectrometer, Orbitrap Exploris 120 (Thermo Fisher Scientific, Bremen, Germany), equipped with a heated electrospray ionization (ESI) source. The analytical column was a HALO 90 Å C18, 2.7 µm, 0.3 × 100 mm (Advanced Materials Technology, Wilmington, DE, USA), connected to a Thermo Scientific Pep Map Neo Trap Cartridge Holder. The heated column compartment was at 35 °C. The mobile phase for the chromatographic separation was mixed from mobile phase A (Chromsystems, reference number 72011) and mobile phase B (Chromsystems, reference number 72002). Gradient elution of the NC pump started at 83% A with a flow rate of 10 µL/min. The flow was linearly decreased from 10 µL/min to 5 µL/min in 2 min. Then, 67% A was programmed from minute 2.0 to 4.7, followed by 63% A until minute 12. From minutes 12 to 14, 100% A was programmed. Finally, from minutes 14 to 16, the initial conditions were set (83% A, 10 µL/min). A loading pump was connected to the LC column flow with a flow rate of 100 µL/min from minute 0 to 0.25 and from minute 15 to 16. From minutes 0.25 to 15, the loading pump was not connected to the LC column, and the flow rate was 10 µL/min. The ion source was operated in both positive and negative ion mode, and the following settings were used: positive ion 3400 V, negative ion 2000 V, sheath gas 8, aux gas 2, ion transfer tube temperature 320 °C. The data were collected both in the full scan and targeted MS2 modes with the following settings. Full scan: resolution 60,000, scan range 100–600 *m*/*z*, RF Lens (%) 70. Targeted MS2 scan: protonated steroid exact masses were selected for MS/MS using 30% normalized collision energy, and the MS/MS scan was set automatically with a resolution of 15,000. Data acquisition and analysis were performed using the TraceFinder 5.1. clinical software (Thermo Fisher Scientific, Bremen, Germany). Figure S1B shows a chromatogram of a urine sample, and Table S1 indicates the retention times and quantitative ions.

### 2.5. Method Validation

#### 2.5.1. Linearity of the Calibration Curves

Linearity was evaluated over a range between 12.5 and 1000 µg/L using six standards. Complete calibration curves were analyzed on 5 separate days for both methods. A 1/X weighted linear regression was used to plot the peak area ratio (steroids relative to IS) versus the corresponding concentration. An isotopic IS was used for each steroid. The slope, y-intercept, and correlation coefficient were calculated for each standard curve. A minimum value of r^2^ = 0.99 was required to pass this validation step. Precision and accuracy versus the nominal concentration of the standards were also calculated. The back-calculated concentrations of the calibrators were acceptable when within ±15% of the nominal values. The lower limit of quantification and the upper limit of quantification were set at the lowest (12.5 μg/L) and highest (1000 μg/L) calibration standard values, respectively [32]. For the LLoQ, accuracy had to be within 100 ± 20% and the RSD below 20% [32].

The analytical responses of the four steroids were assessed to ensure that the calibration curve built in the methanol standards could be used to quantify the urine samples. The slope coefficients (α) of the 6-point steroid-spiked curves in human urine from four different sources were compared with their respective curves in methanol. The response factors (RF) were calculated as α_spiked-urine_/α_methanol_. The back-calculated concentrations of the urine samples with and without RF correction were used to calculate the sum of the absolute values of the relative residuals (C_spiked-urine_ − C_nominal_)/C_nominal_).

#### 2.5.2. Accuracy and Imprecision

To evaluate the accuracy and precision of both methods, back-calculated results of multiple analyses of the two QCs and two urine samples were used. Inter-assay accuracy and precision were calculated on five different days. Accuracy was determined as the difference between the calculated concentrations of the metabolites and the theoretical concentrations of the QCs expressed as percentages following the formula 1 + ((C_obtained_ − C_theoretical_)/(C_theoretical_)). To pass the accuracy test, the mean values had to be within 100 ± 15% of the theoretical value. The imprecision at each concentration level was expressed as the relative standard deviation (%RSD) for each QC and urine sample, which should not exceed 15% [32]. The accuracy and imprecision of the GC-MS method for cortisol measurements were also evaluated according to analysis (*n* = 5) of the human serum cortisol certified reference material ERM-DA192, as this method was shown to be free of the matrix effect in the human serum samples. 

The accuracy of both the GC-MS and LC-HRMS methods was also assessed by adding cortisol, cortisone, 6β-hydroxycortisol, and 18-hydroxycortisol to urine samples and comparing the expected and obtained concentrations with the formula: Accuracy (%) = (C_observed_ − C_expected_)/C_spiked_.

#### 2.5.3. The Selectivity, Carry-Over, and Stability of the Extracts and Comparison between Methods

The selectivity was investigated by analyzing 10 different human urine sources and was indicated by the absence of any endogenous interference at the retention times of the metabolites. The carry-over was evaluated by injecting 2 µL of cyclohexane (GC-MS method) or 3 µL of the mobile phase (LC-HRMS method) immediately after the injection of the higher standard on three separate occasions. The stability of the extracts in the autosampler was evaluated by reanalyzing the QC and urine samples stored inside the autosampler at ambient temperature (GC-MS) or 8 °C (LC-HRMS) for up to 72 h. Finally, ten urine samples were analyzed using both the GC-MS and LC-HRMS methods.

### 2.6. Process Efficiency

Due to the presence of endogenous concentrations in the blank urine, the process efficiency of the LC-HRMS method was assessed by comparing the areas of the ISs in the extracted urine samples (3 individual sources) with the areas of the non-extracted ISs according to the formula: 100 × (A_urine_/A_standard_). Three different concentrations of the ISs were evaluated (12.5, 100 and 250 µg/L).

### 2.7. Cross-Reactivity with Two Common Cortisol Immunoassays

The cross-reactivity of cortisone, 6β-hydroxycortisol, and 18-hydroxycortisol were determined for the Atellica IM1600 (Siemens Healthineers, Erlangen, Germany) and Liaison XL (Diasorin, Saluggia, Italy) cortisol immunoassays. Interference studies were performed in both aqueous solutions without extraction (*n* = 4) and the urine samples extracted with dichloromethane (*n* = 4). Cortisol, cortisone, 6β-hydroxycortisol, or 18-hydroxycortisol were spiked into water or normal human urine (before dichloromethane extraction) at final concentrations of 100, 300, and 500 µg/L. The cross-reactivity of each steroid in the cortisol assays in non-extracted aqueous solutions was calculated using the formula C_spiked steroid_/C_spiked cortisol (100 µg/L)_. The cross-reactivity of each steroid in the cortisol assays in the urine samples was calculated using the formula C_spiked urine sample_ − C_unspiked urine sample_)/C_unspiked urine sample_.

### 2.8. Method Application

The cortisol, cortisone, 6β-hydroxycortisol, and 18-hydroxycortisol concentrations were measured using LC-HRMS in 24 h urine samples (*n* = 60) from 46 patients (23 males, 23 females, 21 to 85 years old) with clinical suspicion of hypercortisolism or with CS in follow-up. Cortisol in urine was also measured using immunoassay (Liaison), and the results were compared with those obtained using LC-HRMS using Deming regression, Bland–Altman, and *t*-student analyses. In addition, linear correlations between cortisol excretion and the excretion of cortisone, 6β-hydroxycortisol, and 18-hydroxycortisol were determined using Pearson’s correlation coefficient (r). A sample with an extremely high urine cortisol excretion (8745 µg/day by LC-HRMS) was excluded from the statistical analyses. Finally, the effect of antisteroidogenic drugs on 11β-hydroxysteroid dehydrogenase and CYP3A4 activities was assessed by calculating the ratios cortisol/cortisone and 6β-hydroxycortisol/cortisol, respectively. The antisteroidogenic drugs included ketoconazole, metyrapone, and osilodrostat, which are used in patients as a medical treatment for hypercortisolism. The concentration results are expressed as means ± SEM, and *p* values were calculated using one-way ANOVA with Tukey’s multiple comparisons test. Statistical analyses were performed using GraphPad Prism 6 (GraphPad Prism Software Inc., San Diego, CA, USA). The study was performed in agreement with the criteria of the Investigation and Ethics Committee of the Hospital Clinic (Barcelona, Spain).

## 3. Results

### 3.1. Linearity of the Calibration Curves

**Figure 1:**
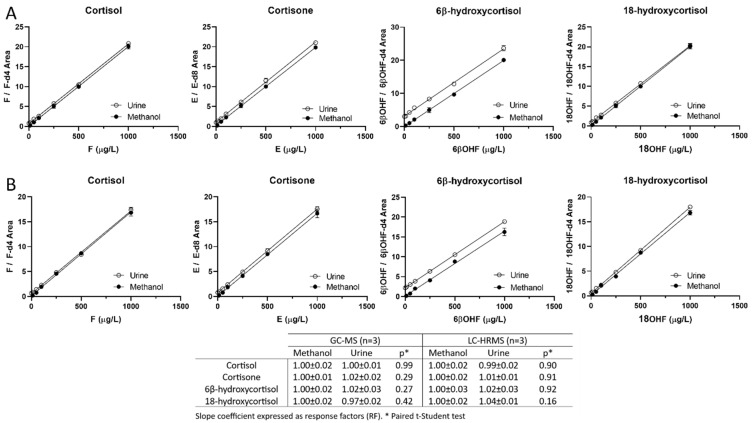
Steroid-spiked curves in human urine compared with the respective curves in methanol measured using the GC-MS (**A**) and LC-HRMS (**B**) methods. F: cortisol, E: cortisone, 6βOHF: 6β-hydroxycortisol, 18OHF: 18-hydroxycortisol. Four replicates were obtained from distinct methanol samples and an additional four from separate serum sources.

For the GC-MS quantification measurements, the SIM areas of each specific ion of the analytes and of the IS were used. Similarly, for the LC-MS quantifications, only the areas of the HRMS target ions (Table S1) were used. The MS/MS scans were not used for the LC-MS quantification. Calibration curves were prepared according to dilution of the working solution in methanol to avoid the potential bias resulting from endogenous steroids being present at different levels in the urine. Both the GC-MS and LC-HRMS methods were linear for the four steroids, obtaining r^2^ values > 0.99. The calibration samples showed an accuracy ranging from 93 to 105% and from 96 to 107%, respectively, for the GC-MS and LC-HRMS methods, and the RSD was <7% (Table 1). Although isotopic ISs were used for each steroid to compensate for any variations during the sample processing, additional validation procedures were necessary to evaluate the appropriateness of the preparation of the calibrations in methanol. Experiments were performed to evaluate the differences in recoveries between the urine samples and methanolic standard solutions. As shown in Figure 1, no significant differences were observed between the slope coefficients (α) of the methanolic solutions and the steroid-spiked curves in urine. The response factors (RF) were calculated as αspiked urine/αmethanol. The implementation of RFs for the samples spiked in urine did not result in better accuracy or precision. Thus, an RF was not necessary despite using a different matrix for the calibration curves and the clinical urine samples. These results support the parsimonious approach of not compensating for different matrices.

### 3.2. Accuracy and Imprecision

The values of the inter-assay accuracy and imprecision of the two QC levels and the values of the inter-assay imprecision of the two urine samples also met the validation requirements and are summarized in Table 2. The lowest calibrator (12.5 µg/L) was chosen for the low limit of quantification. The inter-day accuracy and RSD of the low limits of quantification also met the validation requirement: the accuracy was 93–98% and 97–107%, respectively, for the GC-MS and LC-HRMS methods, and the RSD was <7% (Table 1). In addition, the accuracy and RSD of the cortisol measurements using the GC-MS method evaluated using the human serum certified reference material ERM-DA192 were 96.8% and 5.2%, respectively (Figure S2). Finally, the accuracy was also analyzed by spiking the analytes in human urine samples. The GC-MS method presented mean accuracies ranging from 99 to 107%, obtained by processing three replicates of a urine sample spiked with 125 µg/L of each steroid (Table S2). The LC-HRMS method’s mean recoveries ranged from 91 to 107%, obtained by processing three different urine samples spiked with 12.5 µg/L and 25 µg/L of each steroid (Table S3).

### 3.3. Selectivity, Carry-Over, and Stability of the Extracts and Method Comparison

The analysis of 20 different human urine samples revealed the endogenous presence of the four analytes in all the samples. However, no additional interfering signals were observed. Similar ion ratios were observed between the quantification ions and two other main ions. In addition, there were no carry-over effects after injecting blank samples following an injection of a standard with the highest concentration. The extracts in the autosampler at ambient temperature (GC-MS) or 8 °C (LC-HRMS) were stable for at least 72 h, except for 18-hydroxycortisol measured using LC-HRMS, which was stable for up to 24 h (Table S4). Finally, a comparison of ten urine samples showed good agreement between GC-MS and LC-HRMS for the four steroids without significant bias (Deming’s regression slopes ranged from 0.97 to 1.02).

### 3.4. Process Efficiency

The values for process efficiency were determined by comparing the peak areas of the isotopically labeled ISs between the extracted urine samples and the non-extracted standards. As shown in Table S5, the average values for process efficiency at three different levels were dependent on the amount of steroid present in the urine and ranged from 47% to 75% (cortisol), 47% to 86% (cortisone), 10 to 28% (6β-hydroxycortisol), and 25 to 42% (18-hydroxycortisol).

### 3.5. Cross-Reactivity of Two Common Cortisol Immunoassays

**Figure 2:**
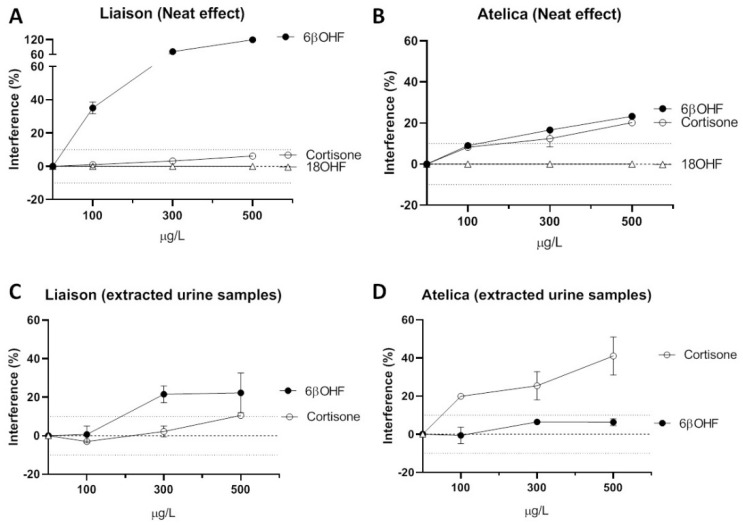
(**A**,**B**) Assessment of interference in urine cortisol immunoassays of varying concentrations of cortisone, 6β-hydroxycortisol (6βOHF), and 18-hydroxycortisol (18OH) standards. (**C**,**D**) Assessment of interference in urine cortisol immunoassays of varying concentrations of cortisone and 6β-hydroxycortisol (6βOHF) following addition of varying concentrations of metabolites to urine samples.

Figure 2 shows the cross-reactivity of the non-extracted standards (in aqueous solution) of cortisone, 6β-hydroxycortisol, and 18-hydroxycortisol with both the Liaison (Figure 2A) and Atellica (Figure 2B) cortisol immunoassays. The Liaison immunoassay presented high cross-reactivity with 6β-hydroxycortisol, lower cross-reactivity with cortisone (<10% up to 500 µg/L), and no cross-reactivity with 18-hydroxycortisol up to 500 µg/L. The Atellica cortisol immunoassay presented significant cross-reactivity with both cortisone and 6β-hydroxycortisol (≈10% at 100 µg/L and ≈20% at 500 µg/L), whereas no-cross reactivity was observed with 18-hydroxycortisol up to 500 µg/L.

The degree of interference was also obtained in a set of urine samples prepared by spiking the same amounts of cortisone and 6β-hydroxycortisol before the extraction of the urine with dichloromethane. The liaison cortisol immunoassay showed significant interference with 6β-hydroxycortisol (cortisol results > 20% in urine samples spiked with 300 and 500 µg/L of 6β-hydroxycortisol), and also with cortisone (cortisol levels 10% higher at 500 µg/L of cortisone) (Figure 2C). In contrast, the Atellica cortisol immunoassay interfered more with cortisone (cortisol levels 20%, 25%, and 41% higher in urine samples, respectively, spiked with 100, 300, and 500 µg/L of cortisone) than 6β-hydroxycortisol (cortisol results 6% higher in urine samples spiked with 500 µg/L of 6β-hydroxycortisol) (Figure 2D).

### 3.6. Method Application

**Figure 3:**
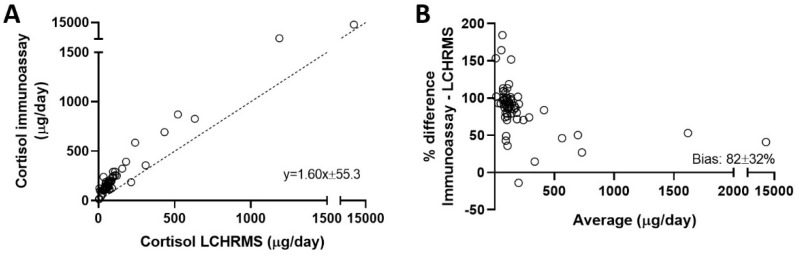
Deming’s regression (**A**) and Bland–Altman bias (**B**) plots of cortisol in urine measured using Liaison immunoassay compared with the LC-HRMS method.

Significantly higher urine cortisol results were obtained using immunoassay (Liaison) in comparison with LC-HRMS, with a mean positive bias of 82 ± 32% (Figure 3). Despite a good correlation coefficient (r^2^ = 0.95), the slope of Deming’s regression line was significantly different from 1 (Liaison = 1.60 × LC-HRMS + 55.3). A paired *t*-test indicated significant differences between the urine cortisol results obtained using immunoassay and LC-HRMS (238 ± 38 vs. 114 ± 24 µg/day; *p* < 0.001).

**Figure 4:**
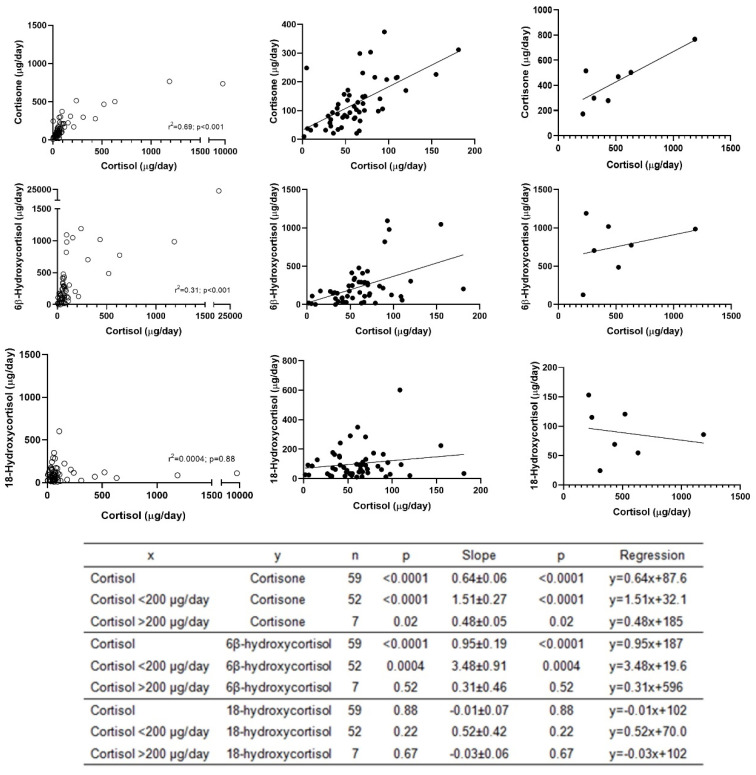
Linear correlation and regression between LC-HRMS measurements of cortisol and cortisone (**top**), 6β-hydroxycortisol (**middle**), and 18-hydroxycortisol (**bottom**) across the whole range, cortisol < 200 µg/day and cortisol > 200 µg/day.

The LC-HRMS cortisol concentrations were significantly correlated with cortisone (r^2^ = 0.69; *p* < 0.001) and 6β-hydroxycortisol (r^2^ = 0.31; *p* < 0.001) in the whole range. However, notable differences were observed in the slope of linear regression when considering high cortisol concentrations (>200 µg/day) or lower concentrations (<200 µg/day). Thus, the slope of the linear regression of cortisol with cortisone lowered from 1.5 to 0.5, and the slope of the linear regression of cortisol with 6β-hydroxycortisol lowered from 3.5 to 0.3 (cortisol < 200 µg/day vs. cortisol > 200 µg/day) (Figure 4). In contrast to cortisone and 6β-hydroxycortisol, no significant correlation was observed between the 18-hydroxycortisol and cortisol concentrations in the urine (Figure 4).

**Figure 5:**
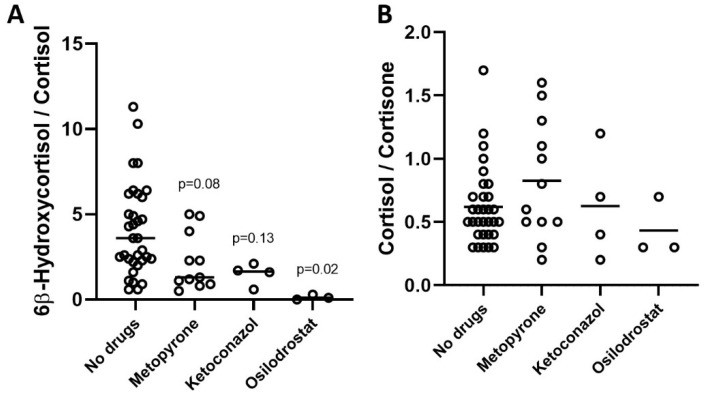
Ratios of 6β-hydroxycortisol/cortisol (**A**) and cortisol/cortisone (**B**) in the urine of patients without antisteroidogenc drugs and on treatment with metyrapone, ketoconazole, and osilodrostat. One-way ANOVA with Tukey’s multiple comparisons test.

The patients treated with antisteroidogenic drugs presented reduced CYP3A4 activity as assessed via the 6β-hydroxycortisol/cortisol urine concentration ratio. The urine of the patients without antisteroidogenic drugs (*n* = 41), with metyrapone (*n* = 12), with ketoconazole (*n* = 4), and with osilodrostat (*n* = 3) presented, respectively, 6β-hydroxycortisol/cortisol ratios of 4.1 ± 0.5, 2.2 ± 0.5, 1.5 ± 0.6, and 0.1 ± 0.1 (Figure 5A). In contrast, no differences were observed between the patients without antisteroidogenic drugs and those treated with metyrapone, ketoconazole, or osilodrostat in terms of the 11β-HSD2 activity, calculated as the ratio cortisol/cortisone, which was 0.6 ± 0.1, 0.8 ± 0.1, 0.6 ± 0.2, and 0.4 ± 0.1, respectively (Figure 5B).

## 4. Discussion

The current study presents an accurate and reproducible LC-HRMS method for the quantification of free cortisol, cortisone, 6β-hydroxycortisol, and 18-hydroxycortisol in urine that is suitable for clinical diagnosis. On account of the high selectivity of LC-HRMS, our method uses only the exact mass (protonated) of each steroid for quantification. In addition, full scan acquisition allows us to obtain the full spectra of the analytes, together with untargeted compounds, at a high resolution, which may be helpful for detecting, for instance, synthetic steroids, the most common cause of CS [33].

The accuracy of measurements of endogenous analytes is challenging. For this reason, different approaches have been proposed to calculating the concentration of the analytes in samples, including the surrogate matrix approach [32]. Our method used liquid–liquid extraction with dichloromethane to yield clean extracts, stable isotope-labeled ISs for each compound, and HRMS *m*/*z* detection. As a result, the comparison between the calibration curves in urine and methanol showed parallel changes across the range of the method, which were linear in terms of the steroid concentrations, covering most of the expected results in CS and non-CS. Therefore, no other surrogate matrices like artificial urine were evaluated. The accuracy, precision, and recoveries of the added steroids also met the current guidelines for analysis [32]. This method includes the use of commercial mobile phases to facilitate routine implementation and the use of micro-LC results with very low volumes of the mobile phase per analytical run (<0.4 mL/sample). However, the mobile phases are part of an LC-MS kit, and their exact chemical composition has not been disclosed.

The current study also presents a GC-MS method for the quantification of the same steroids with high accuracy and precision, which correlates well with LC-HRMS. This GC-MS method is based on the previous GC-MS method developed by Shackleton et al. for the simultaneous quantification of the same four steroids [31]. Although still less straightforward than the LC-HRMS method, there are, however, some differences between our GC-MS method and the one developed by Shackleton et al. that favor its application in routine. Thus, our method takes advantage of the current commercial availability of ISs for each steroid and uses liquid– liquid extraction instead of solid-phase extraction. Both methods use a two-step derivatization procedure to form methyloxime-trimethylsilyl ethers (MO-TMS) to improve the volatility and thermal stability of the four steroids, which contain a combination of hydroxyl and ketonic groups. However, in our method, silylation is achieved using microwave irradiation, which reduces the incubation time from overnight incubation to 2 min. The feasibility and optimization of this microwave-assisted derivatization procedure, and comparison of the derivatization yields for cortisol, cortisone, and 6β-hydroxycortisol under different experimental conditions, have been previously described [34]. Our results on the evaluation of the extract stability in the autosampler (up to 72 h) also support the efficacy of this derivatization approach. Supplementary Table S6 compares the characteristics of the LC-HRMS and both GC-MS methods.

Both urine and serum were free of matrix effects in the GC-MS method, which allowed further evaluation of the accuracy of the cortisol measurements using a certified reference serum material. The Liaison immunoassay grossly overestimated the UFC concentrations compared with GC-MS, as clearly indicated by the Bland–Altman plot (Figure 3B). The inaccuracy of cortisol immunoassays for urine measurements is a well-known analytical limitation [1,2,35,36,37], attributed to cross-reactivity with structurally related metabolites such as tetrahydrocortisol and dihydrocortisol [36]. Therefore, we evaluated whether cortisone, 6β-hydroxycortisol, or 18-hydroxycortisol would also cross-react with two common cortisol immunoassays. The results showed the very high cross-reactivity of the Liaison immunoassay with 6β-hydroxycortisol, which greatly decreased in the extracted urine samples (Figure 2). This may be explained by the higher water solubility of 6β-hydroxycortisol, which results in a lower extraction efficiency in dichloromethane (Table S5). Despite this, the Liaison immunoassay presented cortisol results 20% higher when 6β-hydroxycortisol was added at 300 µg/L. This is a level already found in many normal urine samples since 6β-hydroxycortisol’s normal excretion in urine is 3–15-fold higher than that of cortisol [12,13,31]. Altogether, these results show a significant bias in the Liaison immunoassay due to 6β-hydroxycortisol.

The Atellica immunoassay showed a bias produced by both the unextracted 6β-hydroxycortisol and cortisone standards. The bias produced by 6β-hydroxycortisol highly decreased (<10%) in the extracted urine samples, whereas the bias produced by cortisone remained significant after urine extraction, most likely due to the higher extraction efficiency of the latter (Table S5). Altogether, these results show significant bias in the Atellica immunoassay after dichloromethane extraction due to cortisone and evidence high differences in the cross-reactivities between immunoassays. It should be noted, however, that although mass spectrometry is the gold-standard method for measuring true cortisol, a recent meta-analysis did not observe a higher clinical sensitivity in screening for CS compared to immunoassays [3,4]. It is possible, therefore, that interfering precursors or metabolites of cortisol causing analytical inaccuracy in the immunoassays (cross-reactivity) may contribute to evaluation of the overall adrenal cortisol production.

LC-HRMS measurements of 60 urine samples of patients screened or on follow-up for CS showed good correlations of cortisol with cortisone and 6β-hydroxycortisol but not with 18-hydroxycortisol. In agreement, one sample with an extremely high level of cortisol (8745 µg/day) also presented a very high level of cortisone (737 µg/day) and 6β-hydroxycortisol (22176 µg/day) but not of 18-hydroxycortisol (110 µg/day), which may be related to the more complex metabolism of the latter [16]. The mean cortisol/cortisone ratio was 0.66 at cortisol levels < 200 µg/L and 2.1 at cortisol levels > 200 µg/L, suggesting saturation of the 11βHSD2 enzyme conversion of cortisol into cortisone at high levels of cortisol, as previously observed by Shackleton et al. [38]. A similar pattern was observed with the 6β-hydroxycortisol/cortisol ratio, which suggests a similar enzymatic effect of cortisol excess on 6β-hydroxylation. Finally, although the urine of the patients treated with steroidogenic inhibitors did not show changes in 11βHSD2 activity, a reduction in CYP3A4 activity was observed. Although the causal effect of each drug on CYP3A4 activity cannot be concluded from this study due to the small number of patients and concomitant medications, the results show that patients treated with steroidogenesis inhibitors may present with significant changes in their excretion of free steroid metabolites, as previously observed in a total steroid urine profile [39]. Although the clinical relevance of monitoring these changes in cortisol metabolism should be further assessed, the results point to a considerable impact on the follow-up of these patients if urine cortisol measurements are performed using immunoassay and support the use of mass spectrometry measurements for a proper and comprehensive evaluation of the effects.

## 5. Conclusions

A routine LC-HRMS method was validated for the quantitative measurement of free cortisol, cortisone, 6β-hydroxycortisol, and 18-hydroxycortisol in human urine, which is helpful for evaluating hypercortisolism and other disorders of glucocorticoid and mineralocorticoid metabolism. Simultaneous full scan acquisition allows for additional untargeted high-resolution compound detection of clinically relevant metabolites or drugs. A GC-MS method was also validated for measurement of the same steroids, and significant interference from 6β-hydroxycortisol and cortisone was demonstrated in cortisol immunoassays, which may impact their reliability for monitoring in hypercortisolism medical therapies.

## Figures and Tables

**Table 1:** Inter-day accuracy and imprecision values of the calibration curve standards (*n* = 5 independent calibration runs). A: accuracy; RSD: relative standard deviation.

**GC-MS method**								
	**Cortisone**		**Cortisol**		**6β-Hydroxycortisol**		**18-Hydroxycortisol**	
µg/L	A (%)	RSD (%)	A (%)	RSD (%)	A (%)	RSD (%)	A (%)	RSD (%)
12.5	98.1	4.8	92.8	6.1	93.0	6.4	95.5	5.4
50	100.3	2.2	95.4	2.2	98.5	5.0	97.7	2.2
100	102.4	1.8	103.3	2.5	103.8	4.3	105.4	1.8
250	99.2	2.1	102.1	2.3	101.2	2.6	100.8	1.7
500	100.8	1.6	97.5	1.4	97.3	2.7	101.4	0.6
1000	100.3	1.5	98.8	1.8	101.7	2.3	101.1	0.8
**LC-MS method**								
	**Cortisone**		**Cortisol**		**6β-Hydroxycortisol**		**18-Hydroxycortisol**	
µg/L	A (%)	RSD (%)	A (%)	RSD (%)	A (%)	RSD (%)	A (%)	RSD (%)
12.5	97.2	5.9	104.2	6.1	106.8	6.8	105.3	4.2
50	102.5	4.3	98.3	5.4	95.6	4.1	96.2	3.3
100	104.3	2.7	103.0	4.1	99.1	2.2	101.8	1.9
250	98.5	2.2	98.6	1.2	99.7	2.7	101.3	3.2
500	100.5	1.8	101.2	2.5	101.0	2.1	99.5	1.6
1000	100.2	0.8	99.1	1.0	100.5	2.1	100.7	1.8

**Table 2:** Inter-day accuracy and imprecision values of quality controls and inter-day imprecision values of urine samples (*n* = 5). A: accuracy. RSD: relative standard deviation. QC: quality control.

	**GC-MS**		
	**µg/L**	**A (%)**	**RSD (%)**
**Cortisol**			
QC	25	103.2	3.2
QC	125	112.7	5.1
Urine	58	-	4.2
Urine	184	-	3.7
**Cortisone**			
QC1	25	100.0	5.1
QC2	125	106.5	5.1
Urine	67	-	6.3
Urine	193	-	4.6
**6β-hydroxycortisol**			
QC1	25	100.8	3.9
QC2	125	108.2	4.5
Urine	219	-	6.2
Urine	353	-	8.3
**18-hydroxycortisol**			
QC1	25	99.6	4.8
QC2	125	109.5	5.2
Urine	52	-	7.6
Urine	175	-	5.1
	**LC-HRMS**		
	**µg/L**	**A (%)**	**RSD (%)**
**Cortisol**			
QC	80	98.9	4.0
QC	160	95.8	4.7
Urine	18	-	9.7
Urine	106	-	3.0
**Cortisone**			
QC	80	99.1	4.3
QC	160	97.3	4.2
Urine	40	-	5.6
Urine	126	-	4.4
**6β-hydroxycortisol**			
QC	80	98.0	4.2
QC	160	96.2	3.8
Urine	86	-	5.1
Urine	168	-	2.8
**18-hydroxycortisol**			
QC	80	95.8	7.0
QC	160	93.8	4.7
Urine 1	40	-	10.5
Urine 2	112	-	5.5

## Data Availability

Data is contained within the article or Supplementary Materials.

## Supplementary Materials

The following supporting information can be downloaded at https://www.mdpi.com/article/10.3390/biom14050558/s1, Table S1: Retention times and monitored ions of the analytes and internal standards (LC-HRMS); Table S2: Recovery of added steroids in human urine (*n* = 3), GC-MS method; Table S3: Accuracy of added steroids in human urine (*n* = 3), LC-HRMS method; Table S4: Stability in the autosampler at 8 °C (GC-MS method) and room temperature (LC-HRMS method); Table S5: Efficiency of the extraction procedure; Table S6: Summary comparison of the characteristics of the LC-HRMS and GC-MS methods validated in this study and the GC-MS method published by Shackleton et al. [31]; Figure S1: Chromatogram of urine samples measured using the GC-MS and LC-HRMS methods; Figure S2: (A) Concentration results from the cortisol measurements of the human serum cortisol certified reference material ERM-DA192 using the GC-MS method. (B) Cortisol-spiked curves in human serum (*n* = 3) compared with the respective curves in methanol measured using the GC-MS method.
