# Supplementary material for: LC-HRMS and GC-MS Profiling of Urine Free Cortisol, Cortisone, 6Β-, and 18-Hydroxycortisol for the Evaluation of Glucocorticoid and Mineralocorticoid Disorders
Source: Biomolecules. 2024 May 6;14(5):558. doi: 10.3390/biom14050558 (PMC11117527; doi:10.3390/biom14050558)
Supplement: Supplementary file 1 [file biomolecules-14-00558-s001.zip › biomolecules-2988523-supplementary.pdf]

Supplementary Table S1. Retention times and monitored ions of the analytes and internal standards (LC-HRMS).

Ret time [min]	Compound	Target ion	m/z	Internal standard	m/z
2.2	6β-hydroxycortisol	$[M+H]^+$	379.2115	6β-hydroxycortisol-d4	383.2366
4.7	18-hydroxycortisol	$[M+H]^+$	379.2115	18-hydroxycortisol-d4	383.2366
6.8	Cortisol	$[M+H]^+$	363.2166	Cortisol-d4	367.2417
6.9	Cortisone	$[M+H]^+$	361.2010	Cortisone-d8	369.2512

Supplementary Table S2. Recovery of added steroids in human urine (n=3), GC-MS method.

Steroid	Urine (μg/L)	Added (μg/L)	Obtained (μg/L)	Accuracy (%)
**Cortisol**	59±19	125	183±31	99±7
**Cortisone**	67±25	125	193±10	100±5
**6β-hydroxycortisol**	52±14	125	175±9	98±5
**18-hydroxycortisol**	219±15	125	353±28	107±8

Supplementary Table S3. Accuracy of added steroids in human urine (n=3), LC-HRMS method.

Steroid	Urine concentraton range (µg/L)	Added (µg/L)	Mean recovery (%)
**Cortisol**	30-76	12.5	91
		25	98
**Cortisone**	12-34	12.5	107
		25	88
**6β-hydroxycortisol**	104-192	12.5	96
		25	94
**18-hydroxycortisol**	34-146	12.5	96
		25	96

Supplementary Table S4. Stability in the autosampler at 8ºC (GC-MS method) and room temperature (LC-HRMS method) expressed as accuracy values of quality control and urine samples after 24h and 72 h. QC: quality control.

	GC-MS			LC-HRMS		
	**µg/L**	**24h**	**72h**	**µg/L**	**24h**	**72h**
**Cortisol**						
QC1	26	105.8	113.5	82	96.4	100.2
QC2	138	100.7	112.6	160	100.8	101.4
Urine	58	106.0	112.1	106	106.3	96.9
**Cortisone**						
QC1	26	98.1	101.9	82	105.4	102.9
QC2	130	101.2	104.2	164	100.6	99.5
Urine	70	103.6	108.6	134	100.2	97.1
**6β-hydroxycortisol**						
QC1	26	98.1	96.2	80	100.9	99.3
QC2	135	99.3	100.7	158	102.0	100.9
Urine	224	99.8	100.9	176	96.9	97.7
**18-hydroxycortisol**						
QC1	25	103.9	103.9	76	96.2	89.1
QC2	132	100.0	98.9	164	95.8	84.9
Urine	54	98.1	100.9	106	93.8	78.5

Supplementary Table S5. Efficiency of the extraction procedure (n=3).

	µg/L	Process efficiency (%)
**Cortisol**		
	12.5	75±8
	100	51±6
	250	47±4
**Cortisone**		
	12.5	86±9
	100	53±4
	250	47±7
**6β-hydroxycortisol**		
	12.5	28±4
	100	10±8
	250	10±7
**18-hydroxycortisol**		
	12.5	42±5
	100	30±6
	250	25±5

Supplementary Table S6. Summary comparison of the characteristics of the LC-HRMS and the GC-MS method validated in this study and the GC-MS method published by Shackleton et al (31).

Characteristic	LC-HRMS	GC-MS	GC-MS (31)
Urine volume	0.5 mL	2 mL	5 mL (*)
Urine extraction	Liquid:liquid with dichlorometane	Liquid:liquid with dichlorometane	C18 solid phase extraction with water and methanol
Internal standards	cortisol-d_4, cortisone-d_8 6β-hydroxycortisol-d_4 18-hydroxycortisol-d_4	cortisol-d_4 cortisone-d_8 6β-hydroxycortisol-d_4 18-hydroxycortisol-d_4	cortisol-d_4 cortisone-d_3 cortisone-d_2 (**) 6β-hydroxycortisol-d_2 (**) 18-hydroxycortisol-d_2 (**)
Derivatization	No	methoxyamine hydrochloride (55ºC 60 min), BSTFA (2 min, microwave irradiation)	methoxyamine hydrochloride (60ºC 60 min), BSTFA (16h, 100ºC)
Time of chromatogram	16 minutes	35 minutes	40 minutes
Acquisition	Full scan	Single ion monitoring	Single ion monitoring

(*) Free and total fraction analysis (**) Non commercial

Supplementary Figure S1. Chromatogram of urine samples measured by the GC-MS **(A)** and LC-HRMS **(B)** methods.

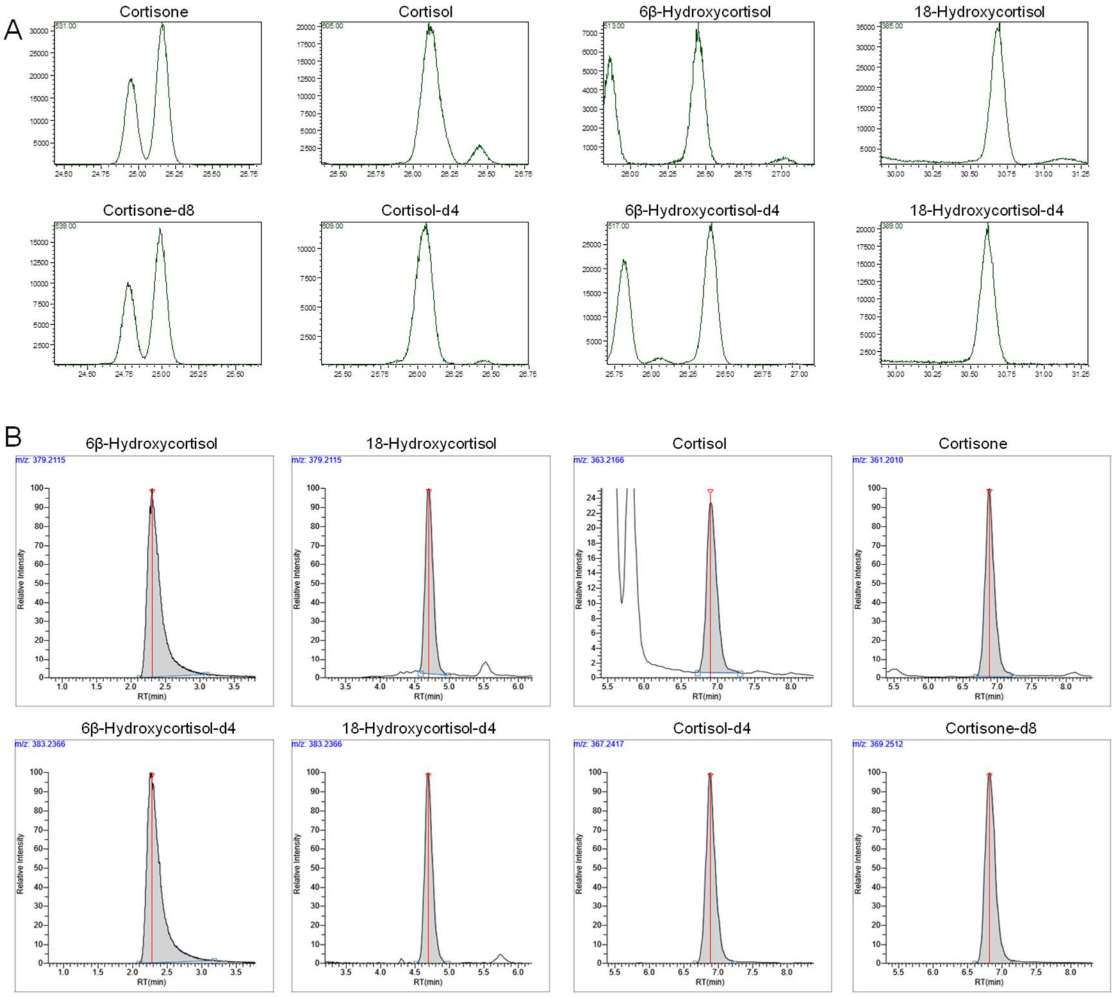

Supplementary Figure S2. **A**. Concentration results of the cortisol measurements of the human serum cortisol certified reference material ERM-DA192 by the GC-MS method. **B.** Cortisol spiked curves in human serums (n=3) compared with the respective curves in methanol measured by the GC-MS method.

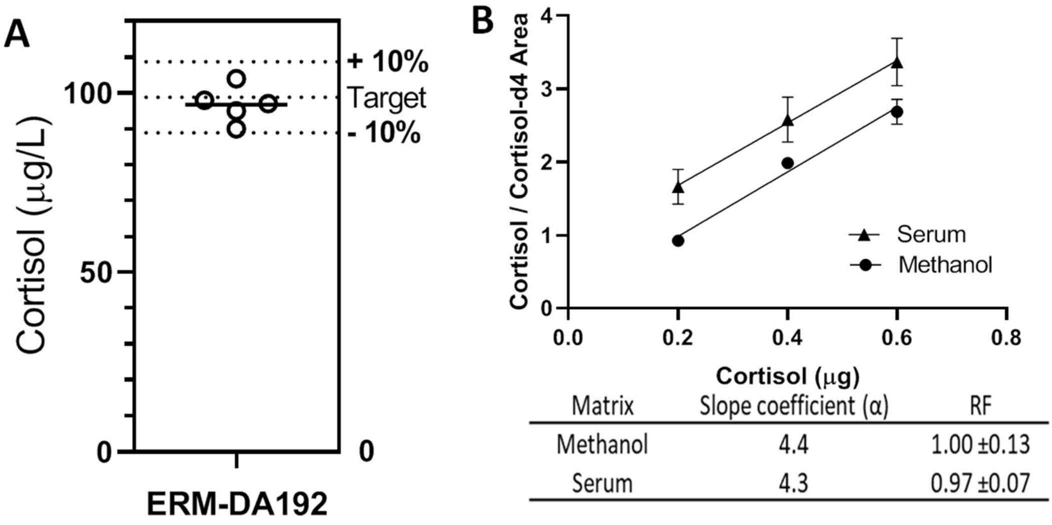

Matrix	Slope coefficient (α)	RF
Methanol	4.4	1.00 ±0.13
Serum	4.3	0.97 ±0.07
